# Spatial smoothing in Bayesian models: a comparison of weights matrix specifications and their impact on inference

**DOI:** 10.1186/s12942-017-0120-x

**Published:** 2017-12-16

**Authors:** Earl W. Duncan, Nicole M. White, Kerrie Mengersen

**Affiliations:** 1 ARC Centre of Excellence for Mathematical and Statistical Frontiers, Queensland University of Technology (QUT), GPO Box 2434, Brisbane, QLD 4000 Australia; 2 Cooperative Research Centre for Spatial Information, Brisbane, Australia; 3 Institute of Health and Biomedical Innovation, Queensland University of Technology (QUT), Brisbane, Australia

**Keywords:** Bayesian inference, Spatial smoothing, Spatial autocorrelation, Spatial weights matrix, Conditional autoregressive model, Markov random field

## Abstract

**Background:**

When analysing spatial data, it is important to account for spatial autocorrelation. In Bayesian statistics, spatial autocorrelation is commonly modelled by the intrinsic conditional autoregressive prior distribution. At the heart of this model is a spatial weights matrix which controls the behaviour and degree of spatial smoothing. The purpose of this study is to review the main specifications of the spatial weights matrix found in the literature, and together with some new and less common specifications, compare the effect that they have on smoothing and model performance.

**Methods:**

The popular BYM model is described, and a simple solution for addressing the identifiability issue among the spatial random effects is provided. Seventeen different definitions of the spatial weights matrix are defined, which are classified into four classes: adjacency-based weights, and weights based on geographic distance, distance between covariate values, and a hybrid of geographic and covariate distances. These last two definitions embody the main novelty of this research. Three synthetic data sets are generated, each representing a different underlying spatial structure. These data sets together with a real spatial data set from the literature are analysed using the models. The models are evaluated using the deviance information criterion and Moran’s I statistic.

**Results:**

The deviance information criterion indicated that the model which uses binary, first-order adjacency weights to perform spatial smoothing is generally an optimal choice for achieving a good model fit. Distance-based weights also generally perform quite well and offer similar parameter interpretations. The less commonly explored options for performing spatial smoothing generally provided a worse model fit than models with more traditional approaches to smoothing, but usually outperformed the benchmark model which did not conduct spatial smoothing.

**Conclusions:**

The specification of the spatial weights matrix can have a colossal impact on model fit and parameter estimation. The results provide some evidence that a smaller number of neighbours used in defining the spatial weights matrix yields a better model fit, and may provide a more accurate representation of the underlying spatial random field. The commonly used binary, first-order adjacency weights still appear to be a good choice for implementing spatial smoothing.

**Electronic supplementary material:**

The online version of this article (10.1186/s12942-017-0120-x) contains supplementary material, which is available to authorized users.

## Background

Consider the problem of mapping disease incidence, prevalence, or mortality with the aim of identifying spatial patterns of the underlying risk surface. Such analyses may identify ‘hot spots’, provide insight into the causal processes, and guide researchers’ efforts in further investigations [1–4]. When analysing spatial data, it is important to account for spatial autocorrelation and sampling variability. Spatial autocorrelation refers to the idea that observations taken at locations near to each other tend to be similar [5–8], while sampling variability refers to differences between areas due to small populations or heterogeneity of individuals within areas, for example [1–4, 9–14]. This is especially true for rare diseases, and when the areal units contain a small population [3, 4].

Numerous statistical models have been developed to address these issues of spatial data. A general overview of some well-known models can be found in [11, 15–19]. Each of these models has the common aim of accounting for spatial autocorrelation and sampling variability so as to satisfy the model assumptions and reduce uncertainty of the estimates. In many disease mapping studies, the modelling approach has been to model the observed data using a Bayesian generalised linear mixed model (GLMM) [3, 20–22], and account for spatial autocorrelation through spatial random effects in the linear predictor. A fairly standard approach is to use a three-stage random effects model: in the first stage, the likelihood for the data is specified by some distribution belonging to the exponential family; in the second stage, the expectation of the response variable is related to the linear predictor through a link function; and the parameters in the linear predictor are assigned prior distributions as the third stage. Examples of this framework can be found in recent papers such as Best et al. [23], Morrison et al. [12], Johnson [24], Pascutto et al. [25], and is also described in Banerjee et al. [26].

Common choices of prior distributions for random effects include the conditional autoregressive (CAR) model [27, 28] and the simultaneous autoregressive (SAR) model [16, 29]. Both models make use of a spatial weights matrix to quantify the relative influence that the random effects have on each other [5, 12, 26, 30]. The effect that the weighting scheme has on the degree of smoothing and the analysis in general has received very little attention in the literature [10]. Some studies have considered multiple weighting schemes, for example [12, 31], but the motivation for doing so is usually to improve model fit and predictive ability. The results from these studies do, however, indicate that different weighting schemes can have a substantial impact on the analysis [10, 12].

The aims of this paper are to (1) review the different specifications of the spatial weights matrix found in the literature; (2) choose a selection of weights matrices for comparison; and (3) use a GLMM with spatial smoothing to analyse a real and synthetic data set with the chosen weights matrices to compare and contrast the effect that they have on model performance and parameter interpretation.

Griffith [32] provides several guidelines for defining the weights, two of which are particularly relevant. The first recommendation is that it is indeed better to apply smoothing than no smoothing at all, which reiterates previous statements about the necessity of smoothing. The second recommendation is that it is generally better to have a small number of neighbours, around 4–6. Getis and Aldstadt [31] add that fewer neighbours is particularly appropriate if the data exhibits spatial heterogeneity. The number of neighbours and assigned weights are often chosen arbitrarily, but more systematic approaches have been proposed. For example, it may be possible to infer a reasonable neighbourhood and weighting scheme from the scientific context [18] or by filtering the spatial effects from the data [32]. Other alternatives include examining the correlogram of relative risks over geographic distance [10] or even using trial and error with respect to the number of neighbours induced [18].

The remainder of this paper is outlined as follows. “Methods” section describes the proposed methods. This includes a detailed specification of a particular CAR model used in Bayesian spatial modelling including prior distributions, several alternate specifications of the spatial weights matrix, and a summary of the two data sets used in the analysis. “Results” section contains the results from the analysis. These results are discussed in “Discussion” section.

## Methods

### BYM model

For rare and non-infectious diseases, the true incidence or number of deaths in a given area is typically estimated by assuming a Poisson distribution,

$$y_{i} \sim {\text{Po}}\left( {E_{i} e^{{\eta_{i} }} } \right),$$

where *y*_*i*_ is the number of observed cases in area $i$, for $i = 1, \ldots ,N$, $E_{i}$ is the expected number of cases, $\eta_{i}$ is the area-specific log-relative risk. $E_{i}$ can be regarded as on offset to account for differences in population, age, and/or risk factors between areas [1, 3, 11, 14, 33]. If data on such characteristics are known, then an alternative is to include these data as additional covariates [11]. Otherwise, $E_{i}$ is generally computed as

1 $$E_{i} = \frac{{\mathop \sum \nolimits_{i} y_{i} }}{{\mathop \sum \nolimits_{i} P_{i} }}P_{i}$$

where $P_{i}$ is the population at risk, which is known as internal standardisation [4, 11, 25, 33]. The area-specific log-relative risk is then expressed as a regression model

2 $$\eta_{i} = \alpha + \beta x_{i} + \gamma_{i} + \varepsilon_{i}$$

where $\alpha$ is the overall fixed effect, $\beta$ is the effect of the spatial covariate $x_{i}$, and $\gamma_{i}$ and $\varepsilon_{i}$ are structured and unstructured spatial random effects respectively. The unstructured spatial random effects are simply the errors which should be independent and identically distributed white noise with unknown variance $\sigma_{\varepsilon }^{2}$ if the spatial autocorrelation is adequately accounted for by the spatial covariate effect and structured spatial random effect [2, 11]. The structured spatial random effects are assumed to have arisen from a Gaussian Markov random field which is consistent with the belief that neighbouring areas have similar spatial effects. This spatial dependency is formalised by imposing the intrinsic conditional autoregressive (ICAR) prior distribution, proposed by [27, 28], on the structured spatial random effects

$$\gamma_{i} |\varvec{\gamma}_{\backslash i} \sim {\mathcal{N}}\left( {\frac{1}{{\mathop \sum \nolimits_{j} w_{ij} }}\mathop \sum \limits_{j = 1}^{N} w_{ij} \gamma_{j} ,\;\frac{{\sigma_{\gamma }^{2} }}{{\mathop \sum \nolimits_{j} w_{ij} }}} \right), \quad {\text{for}}\;i = 1, \ldots ,N$$

where $\varvec{\gamma}_{\backslash i}$ denotes the vector of structured spatial random effects for each area excluding area $i$, and $w_{ij}$ is the element of a symmetric weights matrix ${\mathbf{W}}$ corresponding to row $i$ and column $j$ [11, 15, 33]. This particular model which incorporates the ICAR prior and both the structured and unstructured spatial random effects is often referred to as the BYM model, named after the authors Besag, York, and Mollié [28].

To preserve the identifiability of the random effects, the ICAR prior is constrained by $\mathop \sum \nolimits_{i = 1}^{N} \gamma_{i} = 0$ [4]. However, this only solves the issue of identifiability between $\gamma_{i}$ and $\alpha$; a likelihood identifiability problem still exists because the two spatial random effects are not uniquely identifiable [34]. As a result, the estimate of the structured spatial random effect can become noisy, obscuring spatial patterns. Based on the idea of Eberly and Carlin [34], a simple remedy is as follows: for a posterior sample of size $M$, compute the excess variation (that is, variation not explained by the covariates),

$$\psi = \frac{{{\text{S}}\left(\varvec{\gamma}\right)}}{{{\text{S}}\left(\varvec{\gamma}\right) + {\text{S}}\left(\varvec{\varepsilon}\right)}}$$

where

$${\text{S}}\left(\varvec{\gamma}\right) = sd\left\{ {\mathop {\text{median}}\limits_{m = 1, \ldots ,M} \gamma_{i}^{\left( m \right)} } \right\},$$

and modify the two random effects as follows:

$$\gamma_{i}^{\left( m \right)} \text{ := }\gamma_{i}^{\left( m \right)} - \psi \cdot \varepsilon_{i}^{\left( m \right)}$$

$$\varepsilon_{i}^{\left( m \right)} \text{ := }\varepsilon_{i}^{\left( m \right)} + \psi \cdot \varepsilon_{i}^{\left( m \right)} .$$

for $m = 1, \ldots ,M$. (This modification has been applied to all results for $\varvec{\gamma}$ and $\varvec{\varepsilon}$ throughout this paper.) For the parameters $\alpha$ and $\beta$, weakly informative priors are chosen,

$$\alpha \sim {\mathcal{N}}\left( {0, 100} \right),$$

$$\beta \sim {\mathcal{N}}\left( {0, 100} \right).$$

In accordance with the assumption that the errors are uncorrelated, appropriate prior distributions for the unstructured spatial random effect and associated variance are

$$\varepsilon_{i} \text{ }\sim{ \mathcal{N}}\left( {0, \sigma_{\varepsilon }^{2} } \right),$$

$$\sigma_{\varepsilon } \text{ }\sim{ \mathcal{N}}\left( {0, 10} \right){\mathbb{I}}_{{\left( {0, \infty } \right)}} .$$

A suitable prior distribution for $\sigma_{\gamma }^{2}$ is less straightforward. Earnest et al. [10] caution that the prior for the variance term of the ICAR model can have a noticeable influence on the estimated spatial random effect. Commonly suggested priors for variances or standard deviations, include gamma, inverse gamma, half-Cauchy, and the uniform distribution [10, 11, 35]. If the neighbours are defined appropriately, then the structured spatial random effects for those neighbours should be similar, and therefore the distribution of the variance should have the bulk of the density close to zero. Therefore, a prior which seems consistent with this prior belief is the following gamma distribution:

$$\sigma_{\gamma }^{2} \text{ }\sim{ \mathcal{G}}\left( {3, 1} \right).$$

The model presented so far is deliberately simplistic, as the focus of this paper is about the influence of the weights rather than model utility or complexity. However, this base model can be easily adapted to more complex situations. For example, if additional covariates are available, these can be incorporated as additional terms in Eq. (2). Extensions to spatio-temporal data are also possible, where spatial and temporal smoothing can be employed separately or jointly, depending on the definition of neighbours [11].

### Weights matrix specifications

As mentioned in the previous section, it is common for the weights to be defined as

3 $$w_{ij} = \left\{ {\begin{array}{*{20}l} 1 \hfill & {{\text{if areas}}\;i\;{\text{and}}\;j\;{\text{are neighbours}}} \hfill \\ 0 \hfill & {\text{otherwise}} \hfill \\ \end{array} } \right..$$

The concept of ‘neighbours’ requires further clarification. Often neighbours are defined to be areas which share a common boundary, that is, are adjacent [1, 4, 10–12, 15]. In this case, the weights matrix may be referred to as the first-order adjacency matrix. Note that areas are not considered to be neighbours of themselves, and thus the elements on the diagonal of this matrix are zero by definition [31]. If the areas comprise a regular grid, then the neighbourhood is comparable to restricted forms of the rook and queen chess moves, depending on whether areas which only share a common vertex are considered neighbours [10, 31]. The neighbourhood can be extended to include neighbours of neighbours, resulting in a second-order adjacency matrix, and so on. More generally, we can define an $n{\text{th}}$-order adjacency matrix as

4 $$w_{ij} = \left\{ {\begin{array}{*{20}l} {\omega_{k} } \hfill & {{\text{if areas}}\;i \;{\text{and}}\;j\;{\text{are }}k{\text{th order neighbours}},\quad k =1,..., n} \hfill \\ 0 \hfill & {\text{otherwise}} \hfill \\ \end{array} } \right.$$

where $\varvec{\omega}= \left( {\omega_{1} , \ldots , \omega_{n} } \right)$ is the vector of weights corresponding to each order. Typically, larger weights are assigned to the closest neighbours, so $\varvec{\omega}$ may be defined as a decreasing function of the order, e.g. $\omega_{k} = \exp \left( {\left( {k - 1} \right)/\left( {n - 1} \right)} \right)$. The drawback to these adjacency-based approaches is that they do not account for areas of different sizes. One alternative is to define neighbours as a function of geographical distance. Distance between areas $i$ and $j$ is often measured as the Euclidean distance between their respective centroids [10–12, 18, 24]. Fahrmeir and Kneib [11] point out that using the Euclidean distance implies the assumption of isotropy, that is, the influence between areas $i$ and $j$ is the same in both directions. For the purpose of defining a weights matrix to be used in the ICAR prior, this is actually a desirable property since the weights matrix must be symmetric in order for the structured spatial random effects to yield a Markov random field [11]. Let $\left\{ {d_{ij} } \right\}$ denote these distances. A common definition for distance-based weights is the inverse distance power function:

5 $$w_{ij} = \left( {1/d_{ij} } \right)^{k}$$

for positive integer $k$, with $k$ often taken to be 1 [10, 31]. The larger the exponent $k$, the greater the influence of those areas that are close relative to those further away [10, 31]. Another definition is the exponential decay function

6 $$w_{ij} = \exp \left( { - \lambda d_{ij} } \right),\quad \lambda > 0$$

where $\lambda$ controls the rate of decay [10, 11, 15]. This decay parameter is often taken to be 1, as in Fahrmeir and Kneib [11]. Other authors have suggested more pragmatic approaches to determining this parameter. For example, Earnest et al. [10] recommends setting $\lambda = 10$ based on the autocorrelation of the relative risks. A similar definition is the Gaussian decay function

7 $$w_{ij} = \exp \left( { - \frac{{d_{ij}^{2} }}{{2b^{2} }}} \right),\quad b > 0$$

where the inverse of the bandwidth parameter $b$ determines the rate of decay [15, 19].

Other distance-based weights include the variogram [31, 36], the bi-square, bi-square nearest neighbour, tri-cube, and spherical kernel functions [15, 19, 31], and further definitions are provided in Earnest et al. [10], Dormann et al. [18], and Mugglin et al. [33]. Distance-based weights can also be found in the literature on geospatial models, for example Cressie [16] and Diggle [37]. All these adjacency and distance-based definitions may be collectively referred to as geometric weights. They share the assumption that closer areas have greater influence. However, it is conceivable that areas which are relatively far apart might have a greater influence on each other than areas which are simply nearby geographically. For example, areas with similar covariate values might be expected to have similar relative risk estimates. This yields alternative versions of the distance-based definitions of weights, where the distance $d_{ij}$ is replaced by $\delta_{ij}$, the absolute difference between the covariate values of areas $i$ and $j$:

8 $$\delta_{ij} = \left| {x_{i} - x_{j} } \right|.$$

The smoothing that results from such weights matrices may be still regarded as spatial smoothing since the spatial random effects are smoothed towards the mean value of their neighbours, albeit neighbours which may be far apart geographically. The key difference is that the smoothing is conducted on the covariate space rather than the parameter space. This idea is not new, but it is seldom considered, and very rarely pursued in statistical analyses. For example, Dormann et al. [18] mention that weights matrices can be defined in terms of ‘environmental distance’ as opposed to geographical distance, but only the latter is used in their analysis.

Two references which do actually use smoothing on the covariate space are Kuhnert [38] and Earnest et al. [10]. However, the weights defined in the latter are a function of both the geographic distances and covariate distances, specifically

$$w_{ij} = \frac{1}{{d_{ij} \delta_{ij} }}.$$

Smoothing on the covariate space can be viewed as a more flexible alternative to smoothing on the parameter space for two reasons. First, it relaxes the assumption that the weights are (only) a function of geographic distance. Second, it relaxes the assumption that a large difference in the relative risk between adjacent areas is not possible since these differences are shrunk towards the mean value. For example, if the covariate values corresponding to two neighbouring areas are dissimilar and this is reflected in the weights, then the relative risk estimates for these two areas should potentially be dissimilar too, depending on how accurate the covariate is as a predictor in the model. This second point can be viewed as a simpler alternative to adaptive Markov random fields [11, 39] and areal wombling [40] where the weights are treated as a random variable and therefore allowed to vary.

Aside from a few general recommendations already mentioned, the results from previous studies are not particularly helpful in terms of providing advice on which weighting schemes should be considered for analysis. In fact, the results often suggest conflicting ideas. For example, Morrison et al. [12] found that the weights based on first-order neighbours and four nearest neighbours produced the best model fit, while weights based on second-order neighbours performed worse. Conversely, of the geometric type weights explored by Getis and Aldstadt [31], the ‘rook’ specification which contains at most four neighbours was the least effective, while the ‘queen’ specification improved the model fit. Similarly, Getis and Aldstadt [31] found that the inverse distance power function specification, given by Eq. (5) performs poorly, while Earnest et al. [10] found that the inverse distances were considerably better than the ‘rook’ and ‘queen’ specifications. Both analyses agree, however, that the inverse distance power function appears to perform better when the exponent is 2 compared to the other values tested, namely 1, 3, or 5.

It should be pointed out that the weights matrix ${\mathbf{W}}$ is typically row-standardised such that each row sums to 1 [31]. This helps with interpretation of the parameters and seems to be preferred over global-standardisation [31]. Note that the software used in our analysis automatically row-standardises the weights matrix; the following definitions are the unstandardised versions.

### Study design

Based on the aims of this paper and the recommendations from the literature, 17 definitions of the weights were chosen for the analysis. The first two sets of weights are based on neighbourhood adjacency, specifically first-order neighbours given by Eq. (3) where neighbours are defined as areas which share a common boundary or vertex, and third-order neighbours given by Eq. (4) where $n = 3$, and $\varvec{\omega}= \left( {e^{0} , e^{ - 0.5} , e^{ - 1} } \right)$. The corresponding models are denoted as A1 and A2.

The following distance-based weights are also considered: inverse distance power function given by Eq. (5) for exponents $k$ = 1, 2, and 5, and the decay functions given by Eqs. (6) and (7). Rather than fix the decay and bandwidth parameters at some arbitrary value, these are computed as a function of the mean distance between spatial units, specifically

$$\lambda = \frac{10}{{\mathop {\text{E}}\nolimits_{i,j} \left( {d_{ij} } \right)}}$$

$$b = \lambda^{ - 1} .$$

As noted by Getis and Aldstadt [31], the scale characteristics of data are important. If the decay parameter value was fixed at 10 instead, this would result in a very different weights matrix if the areal units were large administrative regions spanning hundreds of kilometres compared to much smaller areas, including artificial rasters of areas which may be associated with relative distance only. This is also true if the units of geographical distance are changed from kilometres to metres, for example. The justification for the definitions used here is that it alleviates this dependency on the scale of the data and should be applicable to any spatial data set regardless of the scale. The value of 10 in the numerator was determined by trial and error such that for the four data sets analysed, the number of non-negligible neighbours appeared to be fairly consistent for a given model. These distance-based weights models are denoted by D1 through D5. To compare the effect of smoothing on the covariate space, five new models are created by replacing the geographic distances, including those in the calculation of the decay and bandwidth parameters, with the covariate distances given by Eq. (8). These models will be denoted C1 through C5.

The hybrid approach of Earnest et al. [10] is simply the inverse distance specification, where the distance is the product of both the geographic and covariance distances. This approach is certainly not limited to the inverse distance weighting scheme, however. In fact, we also include a hybrid version of each of the five distance-based weights mentioned above, where the distances are replaced by $d_{ij} \delta_{ij}$. The resulting models will be denoted H1 through H5.

The inverse distance power function will produce non-finite values if the distance between two areas is zero. This is theoretically possible for geographic distances, for example when one area is nested within another thus having the same centroid location, but is also applicable to covariate distances, and consequently the hybrid approach. To avoid this issue, Earnest et al. [10] suggest adding a small correction to zero counts. However, the resulting weight will be highly dependent on that arbitrary value. The approach we adopt is to compute the weights without modification, then for each row of the weights matrix, replace the non-finite weights by the maximum finite value in that row. If the graph representing the underlying spatial field is undirected, as is presumed here, then the weights matrix must be symmetric. To retain symmetry, the lower triangular portion of ${\mathbf{W}}$ is replaced by the upper triangular portion of ${\mathbf{W}}^{\text{T}}$.

**Fig. 1:**
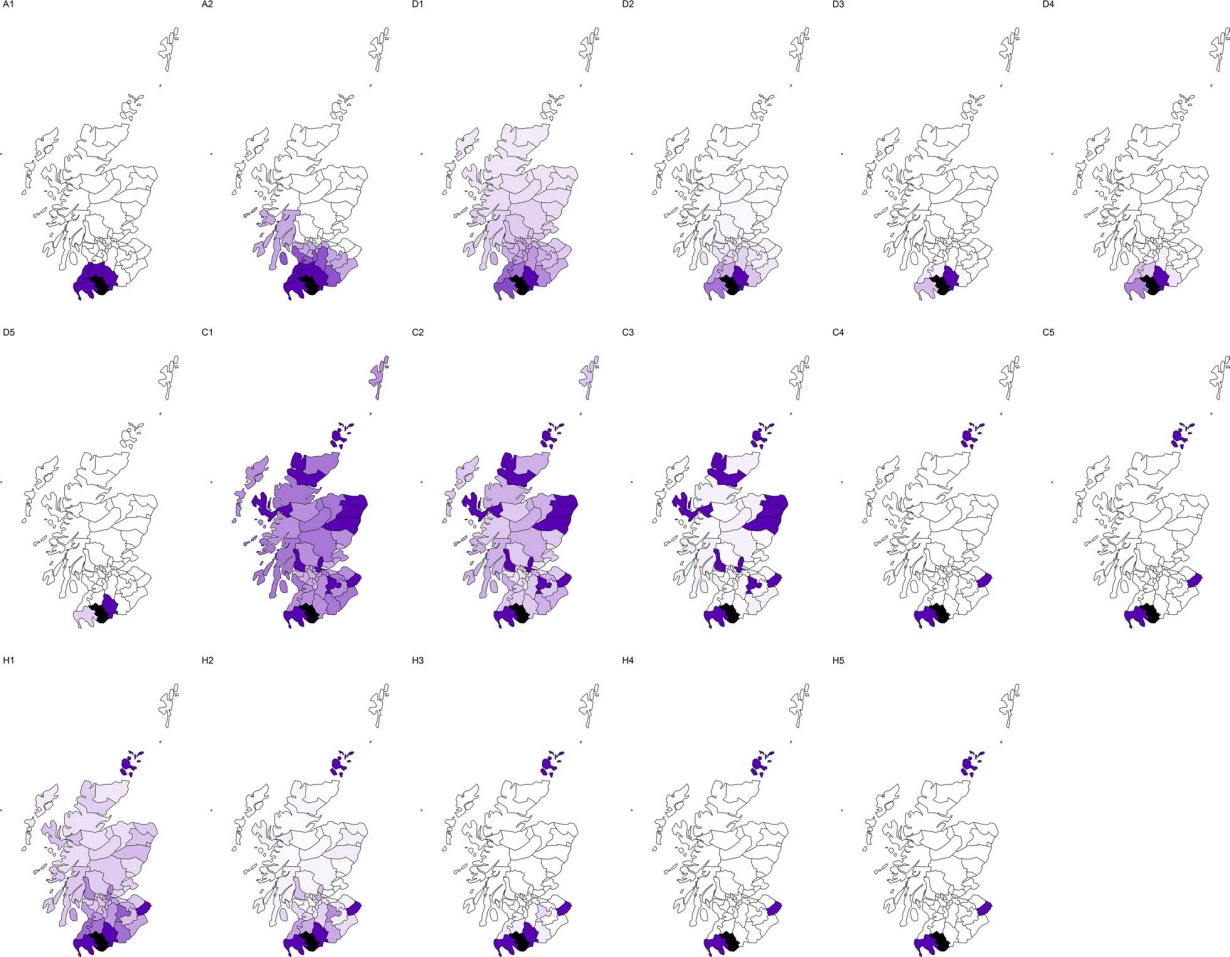
Unnormalised weights for ‘area 43’, shaded black, in the Scottish lip cancer data set. Darker purple areas are neighbours with large weights, signifying a high degree of correlation with area 43, as defined by the weights matrix of the respective models, while white areas have weights very close or equal to zero

**Fig. 2:**
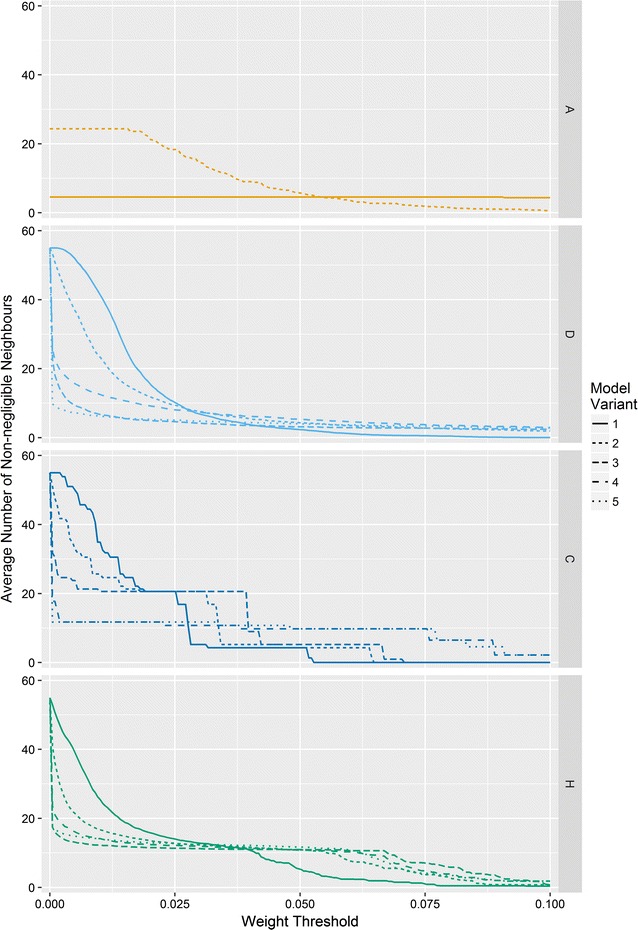
Average number of neighbours excluding areas with normalised weights less than the threshold for the Scottish lip cancer data set

Figure 1 illustrates the differences and similarities of these weighting schemes for the Scottish lip cancer data set, described below. In general, the number of neighbours for each area for all variants of Models D, C, and H will be $N - 1$, albeit the weights will be very close to zero for some neighbours, effectively reducing the dependency to a subset of non-negligible neighbours. The average number of non-negligible neighbours for the Scottish lip cancer data set, as a function of the threshold defining which neighbours are negligible, is shown in Fig. 2. If it is better to have a small number of neighbours as the literature suggests, then it might be expected that weighting schemes which result in a small number of non-negligible neighbours, for a given threshold, perform better.

As a benchmark for the usefulness of including spatial smoothing, a model which does not account for spatial autocorrelation is also included, bringing the total number of models to 18. This model, denoted B, has exactly the same specification except that $\gamma_{i}$ is removed from Eq. (2).

### Data

Four data sets are analysed using the GLMM described above. The first data set is the well-known Scottish lip cancer data set, which has been analysed previously by Breslow and Clayton [20], Rasmussen [22], and Spiegelhalter et al. [41], amongst others. The observed data represents incidence of lip cancer in 56 counties in Scotland. This data set also includes the expected cases which were computed using external standardisation, and a covariate which represents the percentage of the population working in industries that are typically related with high sun exposure, namely agriculture, fishing, and forestry. In both the analysis of Breslow and Clayton [20] and Rasmussen [22], the covariate was scaled by a factor of 10, as is done here.

The other three data sets comprise synthetic data where the spatial units are arranged as a 12 by 12 grid. The observed vales for these three data sets are generated as if arising from (1) a spatial process with no autocorrelation; (2) a random field with strong positive spatial autocorrelation; or (3) a convolution of Gaussian Markov random fields resulting in distinct clusters.

**Fig. 3:**
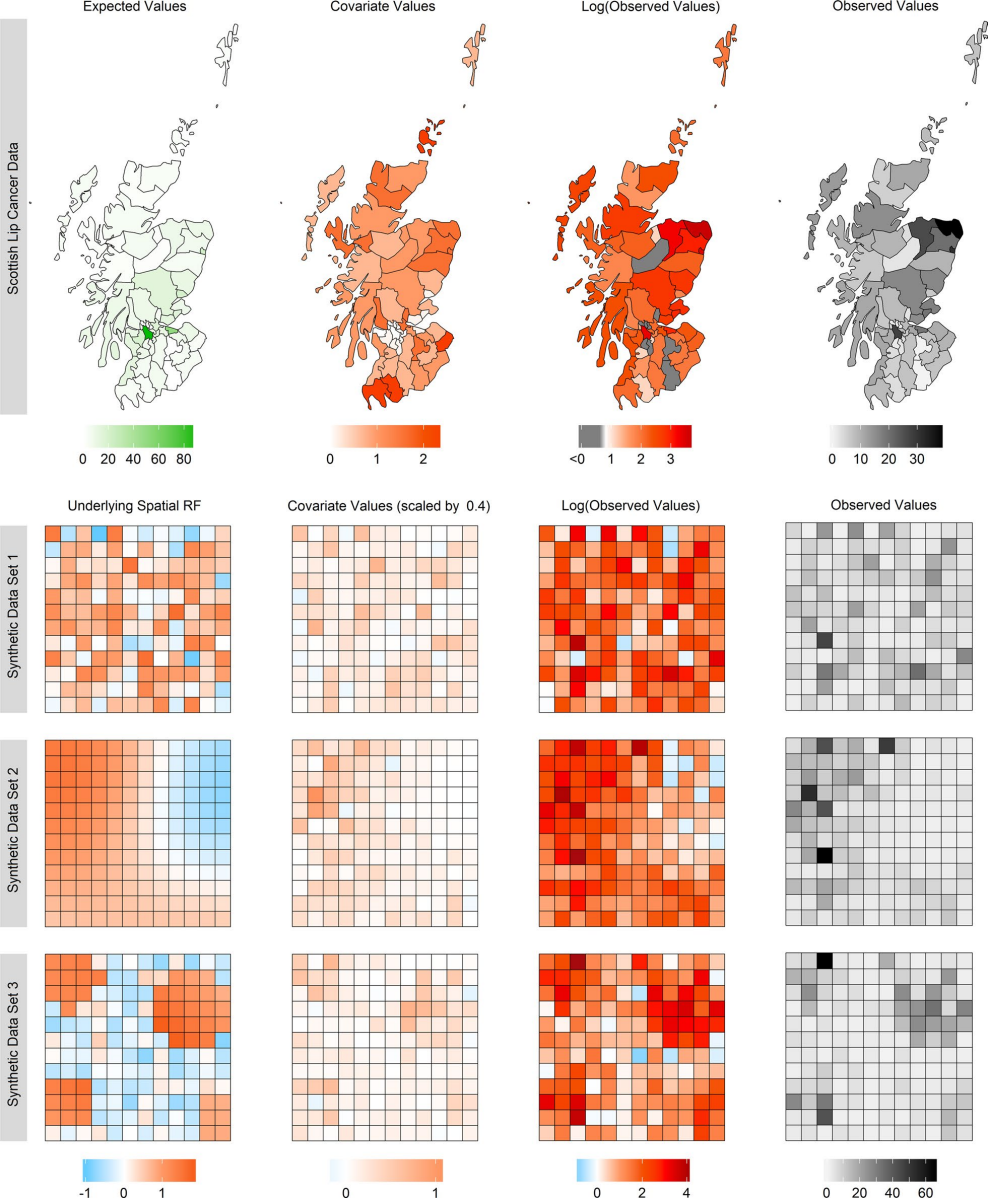
A spatial representation of the expected, covariate, and observed values for the Scottish lip cancer data set, and the main simulated components comprising the generated observed values for the three synthetic data sets

Generating synthetic data for the purpose of comparing how well different models capture spatial autocorrelation is a difficult task. The approach taken here is to decompose the observed values into additive components, generating each of these separately, using the results from the analysis of the Scottish lip cancer data as a guideline for sensible values. The main component is the underlying spatial random field (USRF), which may represent unmeasured covariates. In the first data set, the USRF is simply noise, exhibiting no spatial patterns. In the second data set, the USRF appears as a smooth surface showing a strong degree of autocorrelation, while the third data set contains four clusters of larger, correlated values separated by regions of smaller, noisy values. This was achieved by generating the values from a convolution of two Gaussian Markov random fields, similar to the approach of Devine et al. [42] and Getis and Aldstadt [31]. Next, the covariate values were generated by scaling the respective USRF and adding some noise, thereby attaining some correlation between the two. The reason for this is to facilitate smoothing on the covariate space while allowing enough variation to warrant estimates of both terms in a model. A mixture of a Gaussian and a Gamma distribution was then used to generate the logarithm of the expected counts such that the majority of values were about 2, with few values larger than this, similar to the Scottish lip cancer data set. The observed values are then formed by exponentiating the sum of these components, together with some additional noise. Further details on the generating process can be found in Additional file 1 in the form of R code. The data for the Scottish lip cancer and synthetic data sets are summarised in Fig. 3.

The spatial autocorrelation, or lack thereof, for each data set is perhaps not as obvious as revealed by Fig. 3. The clear patterns of the USRFs generated for the synthetic data sets 2 and 3 are almost completely obscured in the observed data. For the Sottish lip cancer data, whatever spatial autocorrelation might be contributing to the relative risk surface is not apparent from these plots.

Several statistics have been developed for the purpose of quantifying spatial correlation. Moran’s I statistic [43] is commonly used (for example, see Dormann et al. [18]; Getis and Aldstadt [31]; Morrison et al. [12]; Wheeler [19]). This statistic is a function of the weights matrix, however, and different matrices will produce different results. Moran’s I statistic was computed for the observed values in each data set using five different weights matrices, shown in Table 1. Based on the consensus of this statistic under various weight specifications, it would appear that the Scottish lip cancer data is not strongly spatially autocorrelated. The second and third synthetic data sets were generated to exhibit spatial autocorrelation, and this is reflected by Moran’s I. The result for synthetic data set 1 is less clear.

**Table 1:** Moran’s I two-sided p-values for each data set using selected weight specifications defined in the previous section

Weight specification (Model)	Scottish lip cancer	Synthetic data 1	Synthetic data 2	Synthetic data 3
A1	0.1240	0.2619	< 0.0001	< 0.0001
A2	0.5061	0.1114	< 0.0001	< 0.0001
D2	0.2929	0.2109	< 0.0001	< 0.0001
C3	0.6504	< 0.0001	< 0.0001	< 0.0001
H5	0.6923	< 0.0001	< 0.0001	< 0.0001

A p-value close to zero suggests the presence of spatial autocorrelation in the observed data

### Implementation

Each weighting scheme results in a different CAR prior distribution, effectively yielding 18 different models. The model parameters were estimated using Markov chain Monte Carlo (MCMC) sampling, implemented in WinBUGS [44]. The remainder of the analysis was performed using the software R [45]. Two parallel MCMC chains were run for 25,000 iterations with a thinning factor of 5 following a burn-in period of 10,000 iterations. Convergence of the chains was assessed by visual inspection of the posterior distributions and computation of the Gelman–Rubin statistic [46]. The WinBUGS code for the models with and without spatial smoothing are provided in Additional file 2.

### Model evaluation

The Deviance Information Criterion (DIC) [41] is often used as a Bayesian measure of model fit and adequacy, compensating for overfitting by the inclusion of a penalty term. A smaller DIC indicates a better model fit. Following the suggestions of Burnham and Anderson [47] and Spiegelhalter et al. [41], the DIC is used to compare the models in the following way: models with a DIC within 2 units of the ‘best’ model have a similar model fit, while models with a larger DIC have a decidedly worse model fit. In some cases, the DIC defined in Spiegelhalter [41] returns negative values, which are not meaningful. The DIC used throughout this paper is the DIC_3_ variant proposed by Celeux et al. [48] which is more reliable.

However, there are still some concerns about using DIC in a spatial context (see the discussion in Spiegelhalter et al. [41]). As an alternative means of assessing model adequacy, the spatial autocorrelation in the residuals is measured using Moran’s I statistic. If spatial autocorrelation exists within the data, and the model adequately adjusts the log-relative risks, then the residuals ought to be spatially independent in accordance with the model assumptions. To be consistent, the p-values for Moran’s I statistic in the remainder of this paper are based on the binary, first-order weights defined by model A1. Both of these measures were used to assess and compare model fit and adequacy.

Note that in the case of the synthetic data sets, comparison of the posterior estimates of the parameters with the parameters used to generate the data was limited to broad observations about the visual patterns exhibited by the USRF. No attempt was made to compare the numerical values for evaluating accuracy for two main reasons. First, this idea may be possible in simulation studies where the synthetic data can be generated from the model. However, this is not possible with the BYM model because the ICAR prior is a conditional distribution, where the structured spatial random effect depends on its neighbouring values, which do not yet exist. The method of generating the data presented in “Data” section provides the ability to generate spatial random fields with any desired degree of autocorrelation and pattern while avoiding this issue. The second reason is a consequence of the first: the values generated for the USRF are not ‘true values’, and thus by the additive nature of the components comprising the observed values, the concept of true values does not exist for any of the parameters. Nonetheless, the main features of the USRF should be identifiable by a good model.

## Results

### Analysis of the Scottish lip cancer data

**Fig. 4:**
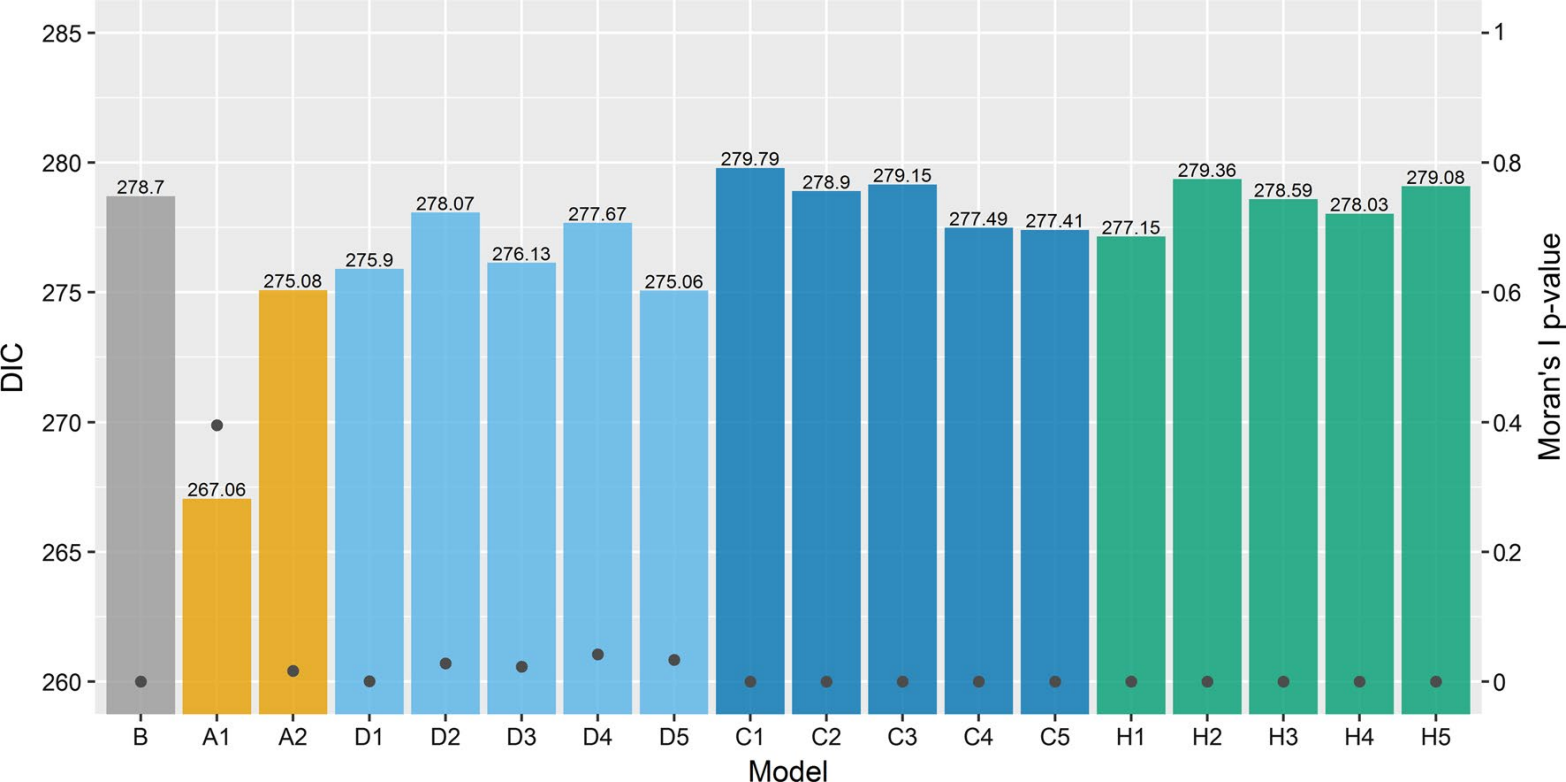
DIC for each model (left axis) overlayed by the two-sided p-values for Moran’s I statistic on the posterior mean of the model residuals (right axis) using the first-order binary weights from model A1

The model evaluation measures for the Scottish lip cancer data set are summarised in Fig. 4. The DIC for each model is shown above each bar. Model A1 performed considerably better, while the remaining models had similar DIC values (all DIC values < 3.7 units of the DIC for model B). The DIC for model B was surprisingly small compared to the models excluding A1, suggesting a reasonable model fit despite the absence of spatial smoothing. However, the models which attempt to account for spatial autocorrelation may provide greater insight than model B, even if the model fit is slightly worse, and interpretation of the other parameters such as the covariate effect may change. This is discussed below. Despite the comparable model fit for most models, Moran’s I statistic, shown on the right axis, suggests that only model A1 accounts for spatial autocorrelation satisfactorily in terms of the model assumptions.

**Fig. 5:**
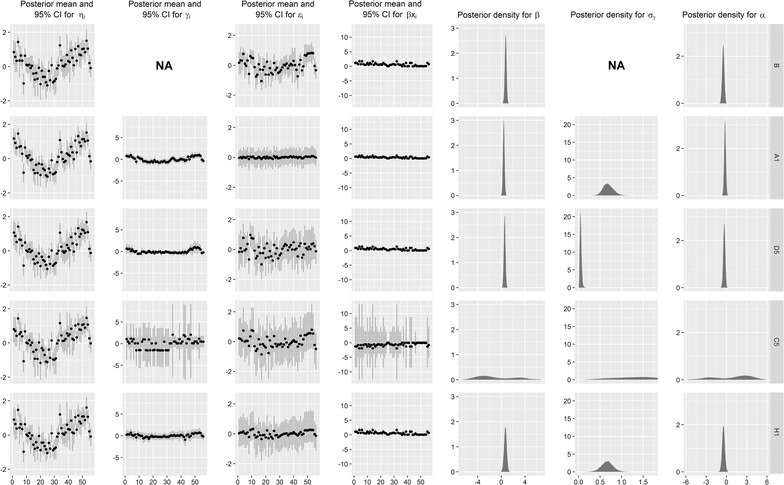
Summary of the parameter estimates based on the MCMC posterior sample for models B, C5, H4, and H5, for a single MCMC chain

**Fig. 6:**
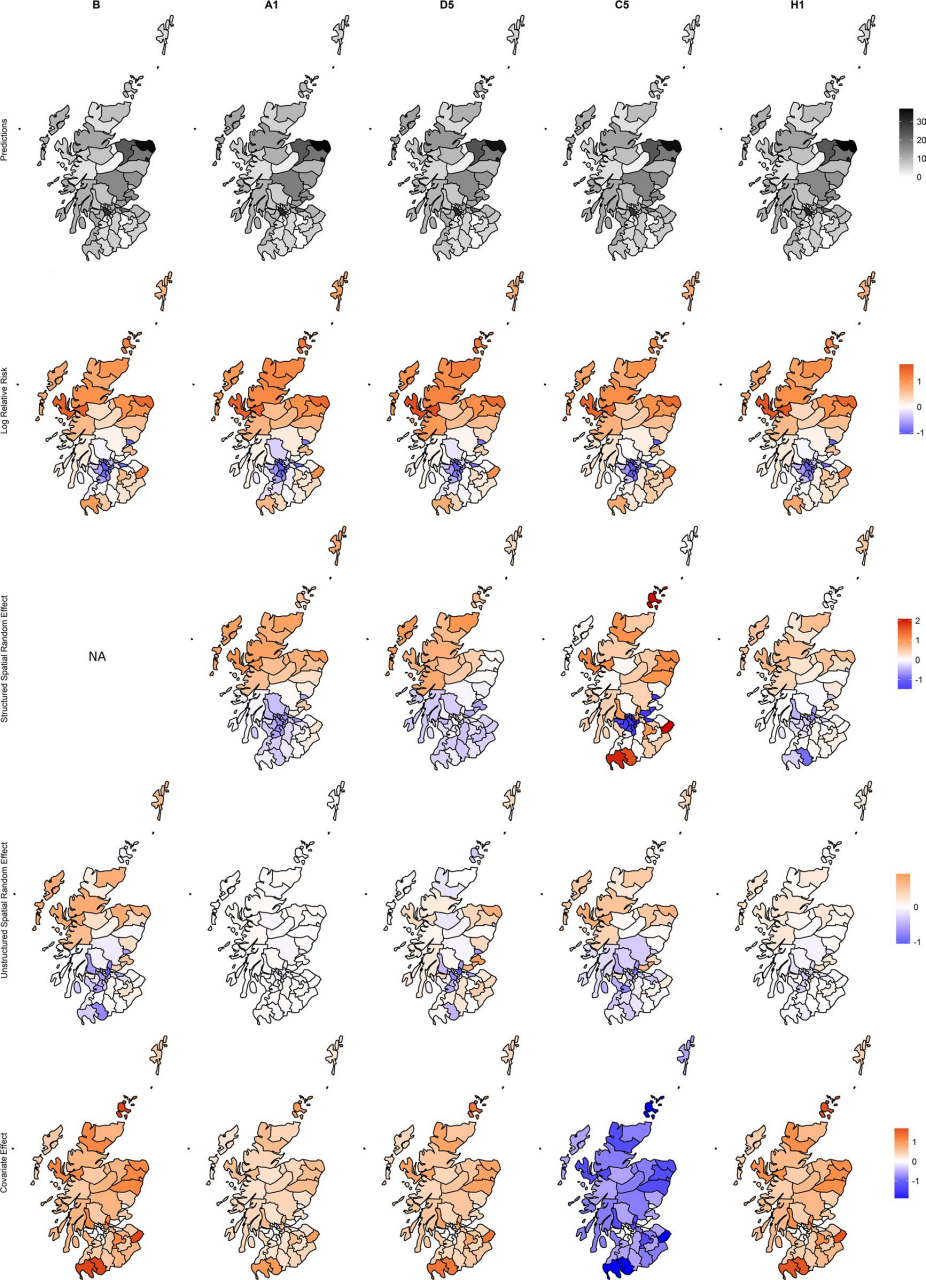
Spatial representation of the predicted values for select models, and a breakdown of the log-relative risk into the main components which comprise it: the structured and unstructured spatial random effects and the area-specific covariate effect, i.e. $\beta x_{i}$. The values are the posterior means. Aside from the plots for the predicted values, the shadings are consistent across all the plots, and the legend shows the range of values for the specific parameter

The posterior estimates of the parameters for the model with the smallest DIC from each class of models are summarised in Fig. 5. The area-specific parameters are ordered in increasing order of the observed values. Aside from model C5, the estimates of the intercept, covariate effect, and variance $\sigma_{\gamma }$ are similar. As expected, the covariate effect is generally positive, indicating that sun exposure is likely an influential factor in lip cancer incidence. The anomalous results of model C5 are difficult to interpret from this figure; these are discussed below when interpreting Fig. 6. While the structured spatial random effect appears quite small in each case, it reduces the unstructured spatial random effect, and provides interesting insights when plotted on a map, as shown in Fig. 6. While the weighting scheme might have a noticeable effect on the individual parameters contributing to the log-relative risk, the log-relative risk appears to be similar for all five models.

Figure 6 shows the spatial representations of the log-relative risk and main model parameters for the models with the smallest DIC from each class of models contributing to this risk surface. The spatial random effects and covariate effect are shown on the same scale as the log-relative risk for ease of comparison. This figure emphasises the contribution of the structured spatial random effect in accounting for spatial autocorrelation. For models A1, D5, C5, and H1, the pattern indicated by the structured spatial random effect suggests that rurality may also be an important risk factor. This spatial random effect is strong enough that the estimated underlying spatial pattern dominates the log-relative risk surface, emphasising the influence of location on relative risk. The covariate effect for model C5 stands out from the other models in that the posterior mean values are negative rather than positive. However, posterior mean values of the intercept term are positive which provide the necessary balance in attaining a reasonable risk surface. While the negative covariate effect may be more difficult or confusing to interpret, the large uncertainty of the estimates (Fig. 5) are probably more concerning.

### Analysis of the synthetic data sets

**Fig. 7:**
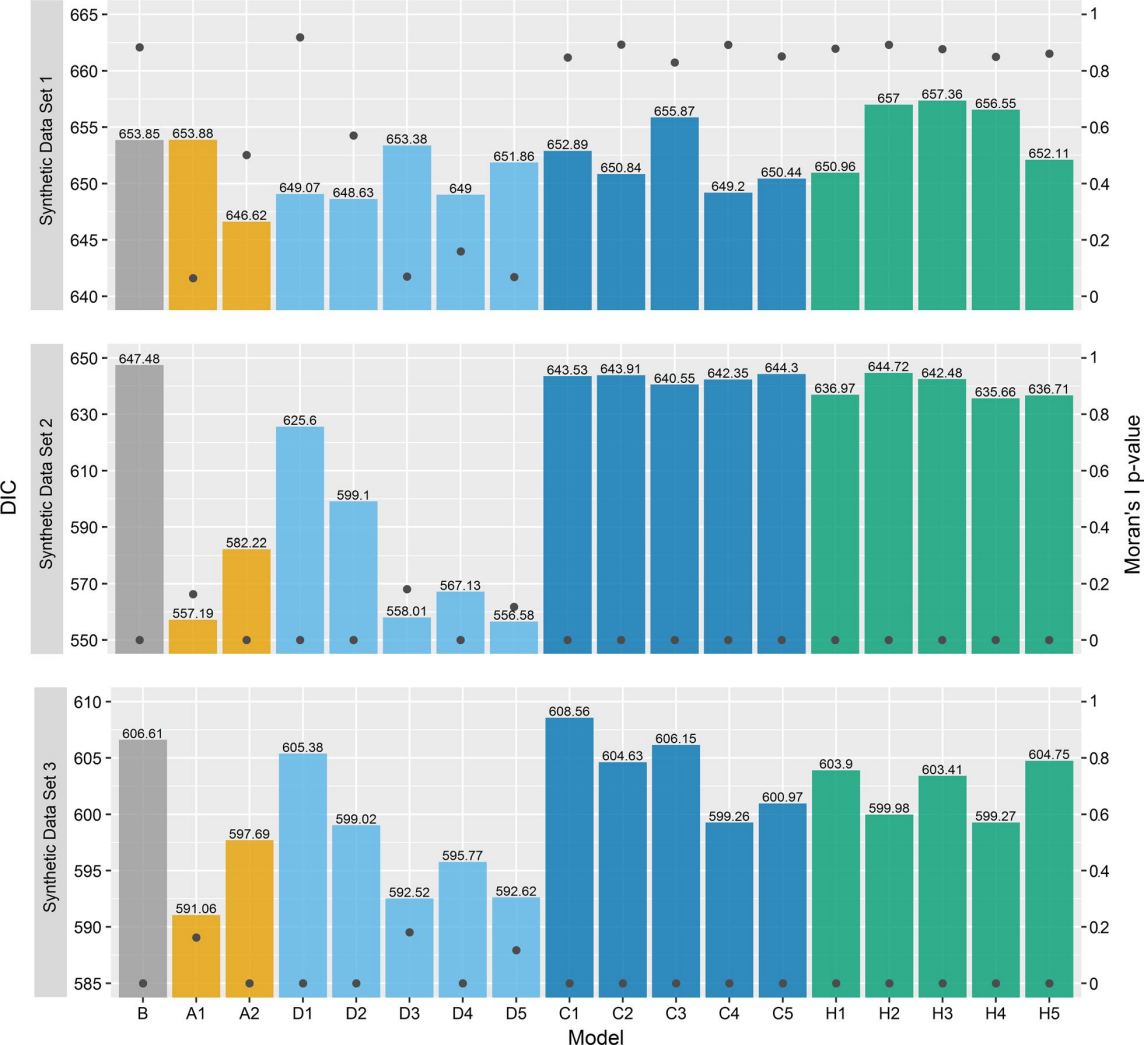
DIC for each model, except model B, across the three synthetic data sets (left axis) overlayed by the two-sided p-values for Moran’s I statistic on the posterior mean of the model residuals (right axis)

The DIC and the p-values for Moran’s I statistic on the model residuals $\varepsilon_{i}$ are provided in Fig. 7. For synthetic data set 1, no spatial autocorrelation was introduced in the data intentionally, and unsurprisingly all of the models achieved a similar model fit. By contrast, model B offers the worst model fit for synthetic data set 2, while all variants of model A and D offer significant improvements (an improvement in DIC of 21.9–90.9 units). Model C and H variants offer smaller improvements in model fit (between 2.8 and 11.8 units), and additionally are more insightful than model B through the estimation of the USRF. Interestingly, only models A1, D3, and D5 appear to remove the spatial autocorrelation from the residuals adequately according to Moran’s I statistic—a result which also holds for synthetic data set 3. Model C and H variants generally perform quite well for this data set, with models C4, C5, H2, and H4 each providing a model fit on par with model D2 (within 1 unit) and better than D1. Given the sharp jumps in the USRF, it is surprising that model C and H variants do not provide an even better fit, and perhaps just as surprising that model A and D variants are able to fit the data so well.

**Fig. 8:**
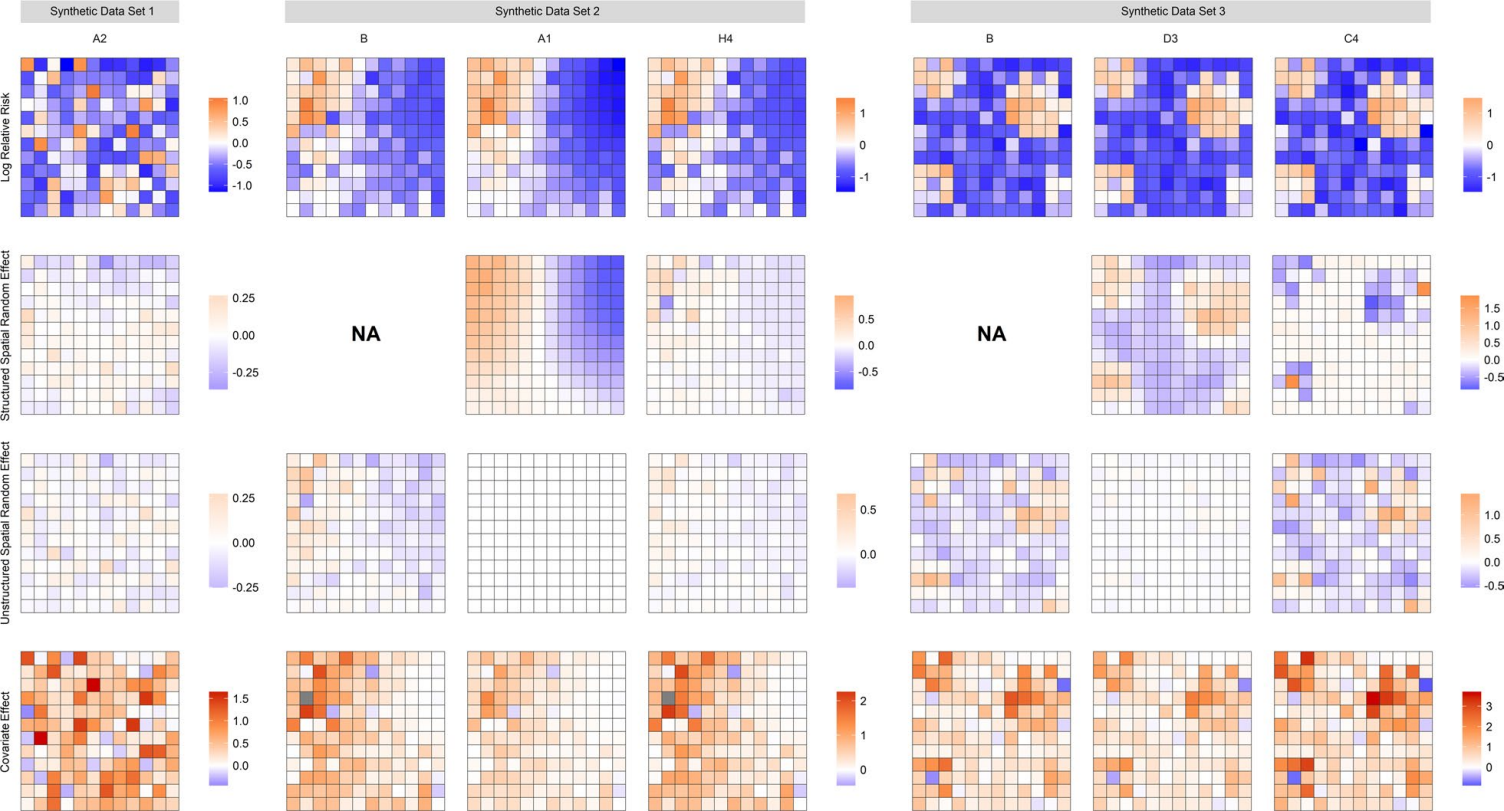
Spatial representation of the log-relative risk surface, and a breakdown of its main components: the structured and unstructured spatial random effects and the area-specific covariate effect, i.e. $\beta x_{i}$. The values are the posterior means. The two areas shaded grey contain values larger than 5 standard deviations from the mean value. The shadings are consistent across all the plots within a given data set, and the legend shows the range of values for the specific parameter

The spatial representations of the structured and unstructured spatial random effects and covariate effect for select models are shown in Fig. 8. For synthetic data set 1, the USRF identified by model A2 is shown. Note that these values are of a similar magnitude as the residuals shown directly below, while the covariate effect is much stronger than either of the spatial random effects. For synthetic data set 2, the USRF is recovered quite well by model A and D variants (only results for model A1 shown). The results for model H4, provided the best fit out of the model C and H variants, are also shown. The USRF for H4 is not represented as clearly or as intensely as model A1. However, the magnitude of the unstructured spatial random effect is noticeably smaller than for model B. For synthetic data set 3, the results were again similar for model A and D variants; the results for D3 are shown here. The structured spatial random effects identify the USRF quite clearly, although the estimates do appear to be oversmoothed. The spatial pattern identified by model C4 is the inverse, with the structured spatial random effect indicating clusters of low values. These values are counter-balanced by the stronger covariate effect, however, resulting in a similar risk surface showing clusters of elevated risk (results not shown here). The results for all parameters and all models are provided in Additional files 3, 4 and 5.

## Discussion

The novelty of the research presented in this paper includes a simple remedy for correcting the identifiability issue between the random effects in the BYM model, and the ideas of covariate and hybrid approaches to spatial smoothing.

Defining the spatial weights matrix based on adjacency or distances between geographic neighbours seems to provide a good model fit regardless of the spatial autocorrelation inherent in the risk surface. In particular, model A1, which epitomises the usual definition of binary, first-order adjacency weights, provided a good model fit for the Scottish lip cancer data, and both synthetic data sets containing a strong USRF. In contrast, the covariate and hybrid definitions of spatial weights generally performed worse. However, the success of these approaches may depend greatly on the correlation between the covariate values and USRF, and for data sets like synthetic data set 3, the jump in values between the clusters and background noise. Overall, it seems that each of the 17 models with spatial smoothing are potentially viable. Given the results from these analyses, and the difficulty in foreseeing what spatial patterns, if any, may be uncovered from a given data set, then choosing a model which incorporates some form of spatial smoothing seems sensible.

Regarding the guidelines from the literature on the appropriate number of neighbours in defining the weights matrix, it appears that models which placed non-negligible weights on only a few neighbours, such as A1, D3, and D5, whether geographic neighbours or neighbours in the covariate space, generally provide a better model fit according to the DIC.

Figures 6 and 8 demonstrate the contribution of the estimated parameters to the log-relative risk for the Scottish lip cancer data and synthetic data sets respectively. Despite the large covariate effects and, in the case of the synthetic data sets, moderate correlation between the covariate and USRF, the inclusion of the structured spatial random effect was able to uncover the USRF in many cases, which not only provided a better model fit than model B, but also offered some insight into the spatial patterns underpinning the log-relative risk surface.

As noted above, there may be several factors required for the success of the covariate and hybrid definitions of the spatial weights. In addition, the multiplicative relationship of the hybrid distances may be underestimating the degree of spatial smoothing required. One alternative would be to use ${ \log }\left( {d_{ij} \delta_{ij} } \right)$ instead, for example. Another potential extension to the work presented in this paper is consideration of multiple covariates. This could lead to new weight specifications and further insight into the impact that they have on spatial smoothing and statistical inference. Analysing larger data sets, and data sets with different spatial patterns may also provide more opportunity to observe differences between these weight specifications, and provide more support for weight specifications like those used in model C and H variants.

Finally, we wish to clarify the notion of smoothing and its effect on the relative risk surface. The term ‘smoothed relative risk’ is often used in the literature, but it should be apparent that the smoothing is applied to only one of the terms contributing to the log-relative risk, at least in the BYM model used here. It would be quite erroneous to think that the estimated risk surface is smooth simply because the structured spatial random effects are smoothed. As Figs. 6 and 8 indicate, the log-relative risk surface still permits adjacent areas with distinctly different values. This may be due to the weights specification which smooths over areas in the covariate space, or because these values are inherited from the other components such as the covariate effect or unstructured spatial random effect. In general, the inclusion of spatial smoothing in the model clearly has a positive impact on estimation of the risk surface, but the extent of smoothing as it applies to the risk surface may be exaggerated. In other words, the issue of oversmoothing that is often attributed to the BYM model does not necessarily imply that the relative risk surface will be oversmoothed.

## Conclusions

In summary, this paper compared 17 specifications of the weights matrix, including adjacency, distance-based, and covariate-based weights. Models using these weights were fit to both data based on simulated risk structures and real data for which the underlying spatial field is unobserved. The effect of the weights matrix on model fit and interpretation of the USRF can be colossal. The commonly used binary, first-order adjacency weights still appear to be a good choice for implementing spatial smoothing. However, using a selection of different definitions of weights may be helpful. Identifying what underlying spatial patterns may be present based on observed data is usually impossible. Therefore, when analysing spatial data, it may be beneficial to account for spatial smoothing, even if the observed data do not appear to be spatially autocorrelated.

## Additional files

**Additional file 1.** Code for generating the synthetic data.

**Additional file 2.** WinBUGS models.

**Additional file 3.** Spatial representations of the spatial model parameters for synthetic data set 1.

**Additional file 4.** Spatial representations of the spatial model parameters for synthetic data set 2.

**Additional file 5.** Spatial representations of the spatial model parameters for synthetic data set 3.

## Abbreviations

BYM: Besag, York and Mollié (model)
CAR: conditional autoregressive
CI: credible interval
DIC: deviance information criterion
ICAR: intrinsic conditional autoregressive
GLMM: generalised linear mixed model
MCMC: Markov chain Monte Carlo
USRF: underlying spatial random field
