# Supplementary material for: Spatial smoothing in Bayesian models: a comparison of weights matrix specifications and their impact on inference
Source: Int J Health Geogr. 2017 Dec 16;16:47. doi: 10.1186/s12942-017-0120-x (PMC5732501; doi:10.1186/s12942-017-0120-x)
Supplement: Supplementary file 1 — Additional file 1. Code for generating the synthetic data. [file 12942_2017_120_MOESM1_ESM.docx]

**S1**: R Code for generating the synthetic data

# Gaussian decay function (Equation (7)): d is the N by N distance

# matrix, and str is a real number representing the relative

# strength of spatial autocorrelation (Gaussian bandwidth

# parameter).

Gaus.decay <- function(d, str){

exp(-0.5 * (d/str)^2)

}

# Generate underlying spatial random field (USRF)

USRF <- matrix(NA, N, 3)

{

# No auotcorrelation

set.seed(3)

USRF[,1] <- rnorm(N, 0.5, sqrt(0.4))

# Random field with moderate spatial autocorrelation

set.seed(26)

Corr <- Gaus.decay(d, 5)

USRF[,2] <- Corr %*% rnorm(N, 0.02, sqrt(0.05))

# Clustered data

set.seed(2)

centre <- c(24, 26, 106, 122) # Centre of clusters

radius <- c(1.5, 1.5, 2.7, 2.1) # Radius of clusters

str <- c(5, 5, 5, 5) # Relative strength of autocorrelation

clust.mean <- c(0.001, 0.015, 0.003, -0.005) # Helps achieve homogeneity

for(k in 1:length(centre)){

Corr <- Gaus.decay(d, str[k])

cluster <- which(d[centre[k],] < radius[k])

USRF[cluster, 3] <- (Corr %*% rnorm(N, clust.mean[k], sqrt(0.01)))[cluster]

}

U.na <- which(is.na(USRF[,3]))

USRF[U.na, 3] <- rnorm(length(U.na), -0.2, sqrt(0.1)) # Background noise

}

# Generate covariate and coefficient

set.seed(2)

x <- matrix(NA, N, 3)

for(k in 1:3){

x[,k] <- (USRF[,k] + 0.7) * rnorm(N, 0.4, 0.3)

}

b <- 0.4

# Generate noise

set.seed(1)

Noise <- rnorm(N, 0, sqrt(0.02))

# Generate [log] expected values

set.seed(5)

log.E <- rnorm(N, 1.4, sqrt(0.25)) + abs(rgamma(N, 1, 2))

# Compute [log] observed values

Int <- -1

log.obs <- USRF + b * x + Noise + Int + log.E

obs <- round(exp(log.obs)) # Convert to integer counts

# Adjust log.E so that maximum obs are < some cap

pop.cap <- 70

too.large <- unique(which(obs > pop.cap, arr.ind = TRUE)[,1])

while(length(too.large) > 0){

log.E[too.large] <- log.E[too.large] - abs(rnorm(length(too.large), 0.2, 0.2))

log.obs <- USRF + b * x + Noise + Int + log.E

obs <- round(exp(log.obs)) # Convert to integer counts

too.large <- unique(which(obs > pop.cap, arr.ind = TRUE)[,1])

}

E <- exp(log.E)
