# Supplementary material for: Spatial smoothing in Bayesian models: a comparison of weights matrix specifications and their impact on inference
Source: Int J Health Geogr. 2017 Dec 16;16:47. doi: 10.1186/s12942-017-0120-x (PMC5732501; doi:10.1186/s12942-017-0120-x)
Supplement: Supplementary file 2 — Additional file 2. WinBUGS models. [file 12942_2017_120_MOESM2_ESM.docx]

**S2**: WinBUGS Models

Model without smoothing:

model {

for (i in 1:N) {

y[i] ~ dpois(RR[i])

RR[i] <- E[i] * exp(eta[i])

eta[i] <- alpha + epsilon[i] + beta * x[i]

epsilon[i] ~ dnorm(0, tau.epsilon)

}

alpha ~ dnorm(0, 0.01)

tau.epsilon <- pow(sigma.epsilon, -2)

sigma.epsilon ~ dnorm(0, 0.1)I(0,)

beta ~ dnorm(0, 0.01)

}

Model with smoothing:

model {

for (i in 1:N) {

y[i] ~ dpois(RR[i])

RR[i] <- E[i] * exp(eta[i])

eta[i] <- alpha + gamma[i] + epsilon[i] + beta * x[i]

epsilon[i] ~ dnorm(0, tau.epsilon)

}

alpha ~ dnorm(0, 0.01)

gamma[1:N] ~ car.normal(adj[], weights[], num[], tau.gamma)

tau.gamma <- pow(sigma.gamma,-2)

sigma.gamma ~ dgamma(3, 1)I(0.00001,)

tau.epsilon <- pow(sigma.epsilon, -2)

sigma.epsilon ~ dnorm(0, 0.1)I(0,)

beta ~ dnorm(0, 0.01)

}
